# A Novel IMU-Based System for Work-Related Musculoskeletal Disorders Risk Assessment

**DOI:** 10.3390/s24113419

**Published:** 2024-05-26

**Authors:** Souha Baklouti, Abdelbadia Chaker, Taysir Rezgui, Anis Sahbani, Sami Bennour, Med Amine Laribi

**Affiliations:** 1 Mechanical Laboratory of Sousse (LMS), National School of Engineers of Sousse, University of Sousse, Sousse 4023, Tunisia; baklouti.souha@eniso.u-sousse.tn (S.B.); abdelbadia.chaker@eniso.u-sousse.tn (A.C.); sami.bennour@eniso.u-sousse.tn (S.B.); 2 ENOVA Robotics S.A., Novation City, Sousse 4023, Tunisia; anis.sahbani@enovarobotics.com; 3 Applied Mechanics, and Systems Research Laboratory (LASMAP), Tunisia Polytechnic School, University of Carthage, Tunis 2078, Tunisia; taysir.rezgui@ept.ucar.tn; 4 Institute for Intelligent Systems and Robotics (ISIR), CNRS, Sorbonne University, 75006 Paris, France; 5 Department of GMSC, Pprime Institute CNRS, University of Poitiers, UPR 3346, 86073 Poitiers, France

**Keywords:** WMSDs risk assessment, wearable technology, inertial measurement units

## Abstract

This study introduces a novel wearable Inertial Measurement Unit (IMU)-based system for an objective and comprehensive assessment of Work-Related Musculoskeletal Disorders (WMSDs), thus enhancing workplace safety. The system integrates wearable technology with a user-friendly interface, providing magnetometer-free orientation estimation, joint angle measurements, and WMSDs risk evaluation. Tested in a cable manufacturing facility, the system was evaluated with ten female employees. The evaluation involved work cycle identification, inter-subject comparisons, and benchmarking against standard WMSD risk assessments like RULA, REBA, Strain Index, and Rodgers Muscle Fatigue Analysis. The evaluation demonstrated uniform joint patterns across participants (ICC=0.72±0.23) and revealed a higher occurrence of postures warranting further investigation, which is not easily detected by traditional methods such as RULA. The experimental results showed that the proposed system’s risk assessments closely aligned with the established methods and enabled detailed and targeted risk assessments, pinpointing specific bodily areas for immediate ergonomic interventions. This approach not only enhances the detection of ergonomic risks but also supports the development of personalized intervention strategies, addressing common workplace issues such as tendinitis, low back pain, and carpal tunnel syndrome. The outcomes highlight the system’s sensitivity and specificity in identifying ergonomic hazards. Future efforts should focus on broader validation and exploring the relative influence of various WMSDs risk factors to refine risk assessment and intervention strategies for improved applicability in occupational health.

## 1. Introduction

Work-related musculoskeletal disorders (WMSDs) present a critical issue within various industries, leading to considerable pain, disability, and financial strains. The global prevalence of these conditions in 2017 was reported at 1.3 billion cases, causing 121.3 thousand deaths and resulting in 138.7 million disability-adjusted life years [1]. Beyond their harmful health effects, WMSDs significantly contribute to reduced productivity and increased healthcare expenses. Indeed, the economic burden of these disorders is profound and widespread. In the United States, for instance, the healthcare expenses associated with WMSDs reached $380.9 billion, amounting to 2% of the GDP in 2016 [2]. The European region showed similar challenges, with costs amounting to €240 billion (2% of GDP) in 2015, attributed to healthcare and productivity losses [3]. This economic strain extends into developing countries as well, where the impact of WMSDs can be particularly acute relative to their worker-based industries and economic capacities. For example, in Tunisia, the costs associated with these disorders represented 0.11% of the country’s GDP in 2021, amounting to 127.8 million TND [4].

Amidst the significant health and economic challenges caused by WMSDs, a range of preventive measures and solutions have been identified and implemented. Traditionally, observation-based and quantitative approaches are commonly used for assessing WMSDs.

Observation-based approaches evaluate ergonomic and health risks by directly observing and gathering self-reported data on workers’ interactions within their work environment. Two commonly used tools are the Rapid Upper Limb Assessment (RULA) and the Rapid Entire Body Assessment (REBA). Both assign scores based on these factors to categorize risk levels and guide intervention needs. Another approach is the Strain Index, which calculates a score based on posture and force to estimate muscle fatigue risk. Additionally, the Rodgers Muscle Fatigue Analysis method focuses on specific risk factors like effort level, how long that effort is sustained, and how frequently it is repeated, providing another lens for evaluating ergonomic risk [5,6]. Meanwhile, quantitative approaches use objective metrics to evaluate ergonomic risks and musculoskeletal disorder hazards, focusing on specific workplace tasks. For instance, the NIOSH Lifting Equation, Manual Handling Assessment Charts (MAC), and Assessment of Repetitive Tasks (ART) are tools designed to measure the physical demands of upper limb tasks.

While traditional methods are valuable, they face several limitations. For example, RULA and REBA might not fully consider factors such as task duration, and recovery times, potentially compromising the accuracy of their scoring systems [7]. Similarly, the Strain Index, despite moderate reliability, raises concerns about consistency in classifying WMSD risks [8]. The Rodgers Muscle Fatigue Analysis method, while ideal for extended tasks, may overlook shorter but impactful fatigue periods [5,6]. The NIOSH Lifting Equation is limited to lifting tasks and assumes ideal conditions [9]. Finally, MAC might necessitate expert analysis for effective interpretation, and ART faces inconsistencies in reliability, especially concerning the evaluation of hand/wrist postures [10,11].

The NIOSH Lifting Equation, focused exclusively on lifting tasks, assumes ideal conditions [9]. The MAC might necessitate expert analysis for effective interpretation, and the ART faces inconsistencies in reliability, especially concerning the evaluation of hand/wrist postures [10,11]. Acknowledging the limitations of the traditional methods, researchers have shifted focus towards integrating advanced technologies. Notably, camera-based systems stand out for their use of video capture and analysis to observe and evaluate human movement and posture for ergonomic risk evaluation. These systems are broadly categorized into two types: marker-based, which requires physical markers for motion tracking, and markerless, which relies on computer vision algorithms for tracking without markers. These systems, while proficient in controlled environments [12,13], exhibit certain limitations. Marker-based systems, despite their precision, require setup and could potentially hinder natural movement [14,15]. In addition, despite the evolution of computer vision algorithms [16,17,18,19], markerless systems often lack the precision required for detailed biomechanical analysis [20,21,22].

Wearable systems, worn directly by individuals, are designed to collect data on human movement, posture, and physiological parameters, for long-term exposure assessment and work training [23]. This technology enables the quantification of angular changes as one body part moves relative to another [24,25,26]. Similarly, innovations in e-textile technology integrated sensory components and conductive fibers within fabric materials, allowing the unobtrusive monitoring of physiological and biomechanical metrics [27,28].

These solutions still face challenges such as user accessibility, data interpretation complexity, and the development of comprehensive risk models [23]. These challenges include constrained application of goniometers [29,30] and scalability/sensor precision challenges in e-textiles [28,31,32,33,34], emphasizing the need for more adaptable, accurate, and user-friendly technologies.

To face these challenges, IMU-based systems emerged as a prevalent choice for the monitoring of human movement and posture. For instance, Alvarez et al. (2015) have successfully applied IMUs for the accurate measurement of upper limb joint angles during occupational tasks, further validating the efficacy of IMUs in ergonomic assessments [35]. Additionally, Zhao et al. (2021) developed an IMU-based system tailored for construction workers, facilitating early identification of WMSD risk factors through comprehensive posture assessment [36].

Despite their advantages, IMU-based systems require the precise calibration and interpretation of raw sensor data for accuracy [37,38,39,40,41]. Environmental and personal factors, such as electromagnetic interference, pose challenges to data quality and joint monitoring accuracy, requiring thorough consideration during implementation [42,43,44].

This paper introduces a wearable IMU-based system aimed at objectively assessing WMSDs, capturing three-dimensional upper body movement and orientation data via eight IMUs. This approach avoids the need for magnetometer readings by incorporating calibration strategies and algorithms for orientation data based on gyroscopes and accelerometers. Furthermore, analytical algorithms were developed to identify ergonomic risks from data patterns, offering objective metrics and visualizations for precise risk identification and intervention assessment. Emphasizing user-friendliness and adaptability, the system ensures minimal interference with workers’ activities and comfort, complemented by an interface that simplifies monitoring and analysis processes for medical experts.

## 2. IMU-Based Monitoring System

### 2.1. System Design

**Figure 1:**
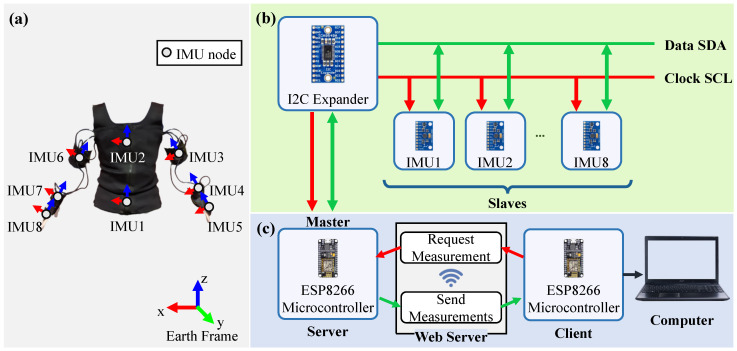
IMU-based Wearable WMSDs Risk Assessment System: (**a**) System Overview, (**b**) Inter-Component Data Circulation, (**c**) Data Flow Between Components and Computer.

The wearable system is designed using eight IMU sensors (MPU9250) (Figure 1a) wired to a microcontroller (ESP8266) through an I2C communication protocol expander (Figure 1b) [45].

This system acquires data from the sensors at a rate of 10 Hz. The microcontroller system acts as a server for the sensors, communicating wirelessly via an asynchronous web server with a client microcontroller (ESP8266) linked to a secure computer (Figure 1c). This enables efficient, non-blocking communication, allowing the server to handle real-time data from multiple sensors simultaneously [46].

The eight IMU sensors were placed on the wearer’s upper arms, forearms, hands, upper back, and lower back, aligning with anatomical axes for optimal data capture [47,48]. Neoprene straps and scratch were used to comfortably secure the IMUs to the upper limbs, while a custom-designed vest-like tank with specific attachment points housed the lower back IMUs, the integrated circuit board, and the power source. This ensured both participant comfort and data collection accuracy.

This study solely focuses on gyroscope and accelerometer measurements to overcome potential magnetic interference from machinery [44]. The IMU sensor data, including elapsed time, acceleration, and gyroscope readings, were collected at a 10 Hz sampling rate using a custom-built Matlab application. The management of the system is facilitated through a user-friendly interface that features various tools including reporting participant information, recording data in real-time, and calibrating sensors. Additionally, it provides functionalities for estimating sensor orientation, calculating upper-limb joint angles, and estimating effective WMSD risk, enhancing the system’s applicability and efficiency in data processing.

### 2.2. Sensor Calibration and Fusion for Orientation Estimation

Before being tested, each sensor was calibrated with initial gyroscope and accelerometer biases. A tilt correction was also applied to the sensor output to account for inaccuracies in the orientation of each sensor node relative to the global frame, particularly the earth frame where *z*-axis is represented by the gravity vector g. Leveraging Earth’s gravity vector as a reference (earth frame) [49], research has shown the feasibility of estimating orientation using accelerometer data alone [50,51]. This estimation relies on a correction factor that accounts for the relative orientation between the measured accelerometer readings and the gravitational vector established through a static position. In this context, the orientations with respect to the X-axis ($\theta_{a}$), Y-axis ($\psi_{a}$), and Z-axis ($\phi_{a}$) can be determined using Equations (1)–(3).

(1) $$\theta_{a}=\tan^{-1}\left(\frac{A_{\mathrm{ref},x}}{\sqrt{A_{\mathrm{ref},y}^{2}+A_{\mathrm{ref},z}^{2}}}\right)$$

(2) $$\psi_{a}=\tan^{-1}\left(\frac{A_{\mathrm{ref},y}}{\sqrt{A_{\mathrm{ref},x}^{2}+A_{\mathrm{ref},z}^{2}}}\right)$$

(3) $$\phi_{a}=\tan^{-1}\left(\frac{\sqrt{A_{\mathrm{ref},x}^{2}+A_{\mathrm{ref},y}^{2}}}{A_{\mathrm{ref},z}}\right)$$

A Kalman Filter for sensor fusion of gyroscope and accelerometer data is implemented. The filter was developed internally and utilizes correction matrices that are identified and adapted specifically for our system. The filter initializes with sensor data and noise models and then progresses through prediction and correction stages. In prediction, gyroscope data forecast orientation changes, and the state is updated using transition models. The correction stage refines these predictions by incorporating accelerometer measurements.

### 2.3. Joint Angles Estimation

**Figure 2:**
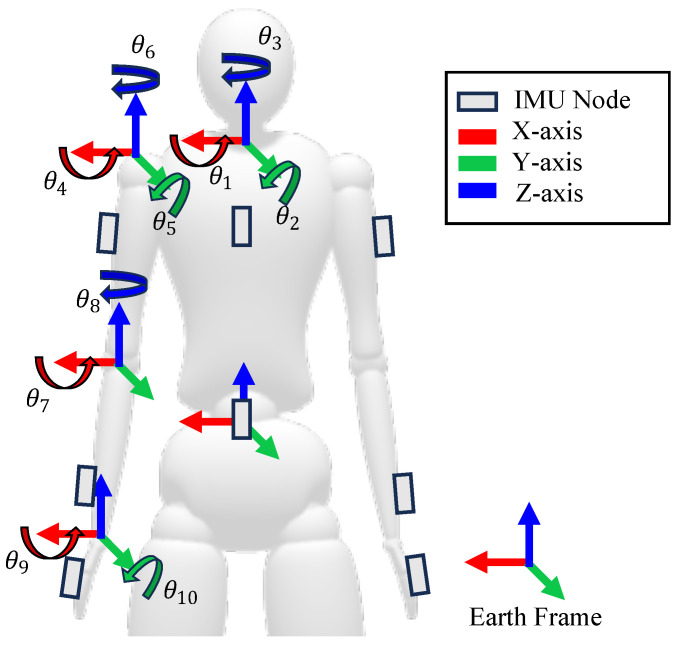
Representation of Upper Body Joint Angles with Local Anatomical Coordinate Systems Defined by Bony Landmarks Coinciding with IMU Sensor Frames Aligned with the Earth Frame.

As shown in Figure 2, the upper body’s movement can be modeled as a combination of 10 degrees of freedom (DOF): 3 DOF in the trunk (flexion/extension, lateral rotation, and rotation), 3 DOF in the shoulder (flexion/extension, adduction/abduction, and internal/external rotation), 1 DOF in the elbow (flexion/extension), 1 DOF in the forearm (pronation/supination), and 2 DOF in the wrist (flexion/extension and radial/ulnar deviation).

In this study, quaternions were employed to estimate the joint angles and to prevent the problem of gimbal lock [52,53,54]. For each IMU, the orientation is represented as a quaternion q. These quaternions are expressed in the earth frame and are denoted as follows: $q_{\mathrm{LB}}^{E}$ for the lower back, $q_{\mathrm{TR}}^{E}$ for the trunk, $q_{A}^{E}$ for the upper arm, $q_{F}^{E}$ for the forearm, and $q_{H}^{E}$ for the hands.

The relative orientation $q_{\mathrm{relative}}$ between two IMUs, representing adjacent body segments, is computed using quaternion multiplication to derive joint angles, as shown in Equation (4). Here, $q_{\mathrm{IMU}1}$ and $q_{\mathrm{IMU}2}$ are the quaternions representing the orientations of the two adjacent IMUs, and $q_{\mathrm{IMU}1}^{-1}$ is the inverse of $q_{\mathrm{IMU}1}$.

(4) $$q_{\mathrm{relative}}=q_{\mathrm{IMU}2}\times q_{\mathrm{IMU}1}^{-1}$$

To convert the quaternion representation to more interpretable joint angles, the Euler representation is used. The conversion prioritizes the joint with the expected largest range of motion (ROM) for improved numerical stability [53]. Typically, the most significant range of motion for upper body joints occurs during flexion and extension movements [55]. Nevertheless, the shoulder joint may exhibit its largest range of motion during abduction or adduction [55]. Consequently, the calculation of joint angles was conducted adhering to these considerations, as depicted in Table 1, where $q_{A}^{B}$ is the inverse of $q_{B}^{A}$.

Following the Euler sequences defined in Table 1, the relative quaternions are converted into joint angles. For example, the trunk has a relative quaternion $q_{LB}^{TR}=\left[q_{x},q_{y},q_{z},q_{w}\right]$ and follows an XYZ sequence. Therefore, the trunk joint angles $\theta_{1}$, $\theta_{2}$, and $\theta_{3}$ are calculated as given by Equations (5), (6), and (7), respectively.

(5) $$\theta_{1}=\arctan2\left(2\left(q_{w}q_{x}+q_{y}q_{z}\right),1-2\left(q_{x}^{2}+q_{y}^{2}\right)\right)$$

(6) $$\theta_{2}=\arcsin\left(2\left(q_{w}q_{y}-q_{z}q_{x}\right)\right)$$

(7) $$\theta_{3}=\arctan2\left(2\left(q_{w}q_{z}+q_{x}q_{y}\right),1-2\left(q_{y}^{2}+q_{z}^{2}\right)\right)$$

## 3. Quantitative Modeling of WMSDs Risks Based on IMU Data

### 3.1. Risk Factors Evaluation

This study investigates three principal risk factors contributing to WMSDs in modern work environments: exertion exposure, postural load, and exertion level, as identified by Kumar’s foundational work [56,57]. The exertion exposure includes the duration, frequency, and recovery time associated with static and dynamic postures; postural load relates to the body’s positioning during tasks, and the exertion level corresponds to the physical effort demanded by the task. These risk factors are evaluated using a combination of data collected from the wearable IMU system and participant self-reporting.

Exertion exposure and postural load are quantified with the IMU system. Static postures, assumed in this study as joint angles not varying more than 20% of the joint range of motion from a maintained position, are assessed by comparing their Constant Work Duration (CWD) to the Endurance Time (ET), defined by international standards [56,57,58,59,60].

Job frequency is determined by counting job cycle repetitions within a set period and assessing their duration, using Constant Frequency (CF) from the IMU-based system, Preferred Job Frequency (PF), and Maximum Frequency (MF), with ergonomic guidelines suggesting frequency >10 repetitions/min or job cycle duration <30 s as high, recommending two repetitions/min as optimal [56,57,59,61,62,63].

Postural load is determined using IMU data reflecting upper body Motion Requirements (MRQ) during tasks. Postural load, evaluated through the IMU system, compares the observed Motion Requirements (MRQ) with safe Mid-Range Values (MDR) and joint limits denoted by Extreme motion values (E), based on ergonomic standards [58,59,64].

Conversely, exertion level and recovery time assessments rely on participant self-reports. Exertion levels are gauged using the BORG CR10 scale, comparing reported Constant Work Levels (CWL) with the research-defined Preferred Work Level (PWL) (≈3–4 on BORG CR10) and Maximal Voluntary Contraction (MVC) [56,57,65,66].

Recovery time is assessed by comparing the Allowed Rest (AR) recorded from participant responses and work schedules, to Required Rest (RR) (10–20% of work time or 5–10 min/h) which is the minimum rest for low risk [67,68,69].

### 3.2. Linking Risk Factors to WMSDs Risk Scores

Building on the collected IMU data and self-reported information, risk models were developed to link the aforementioned risk factors to their respective risk scores. For instance, static postures are known to lead to muscle fatigue and discomfort the longer they are held [70,71,72,73,74]. In our study, the risk associated with static posture duration (R1) is quantified using Equation (8).

(8) $$R_{1}=\left\{\begin{array}{cc} \frac{CWD}{ET}, & \mathrm{if}\;CWD\in [0,ET]\;\mathrm{and}\;ET>0\\ 1, & \mathrm{if}\;CWD>ET\;\mathrm{or}\;ET=0 \end{array}\right.$$

Similarly, tasks performed at high frequencies elevate the risk of WMSDs [56,57,59,61,62,63]. The risk related to job frequency (R2) is expressed using Equation (9).

(9) $$R2=\left\{\begin{array}{cc} 0, & \mathrm{if}\;CF<PF\\ \frac{CF-PF}{MF-PF}, & \mathrm{if}\;CF\in [0,MF]\\ 1, & \mathrm{if}\;\mathrm{Cycle}\;\mathrm{duration}\leq 30\;s,\;\mathrm{or}\;CF>MF \end{array}\right.$$

Furthermore, maintaining proper posture is crucial for musculoskeletal health, as evidenced by established “comfort angles” and ranges [58,59,64]. Deviating from these comfortable postures increases the likelihood of WMSDs, underscoring the importance of maintaining mid-range joint angles [56,57]. Our study quantifies the risk associated with postural load (R3) through Equation (10).

(10) $$R3=\left\{\begin{array}{cc} \frac{MDR-MRQ}{MDR-E}, & \mathrm{if}\;MRQ<0\\ 0, & \mathrm{if}\;MRQ\in \mathrm{mid}\;\mathrm{range}\\ \frac{MRQ-MDR}{E-MDR}, & \mathrm{if}\;MRQ>0 \end{array}\right.$$

Additionally, the existing literature highlights a direct link between workers’ perceived exertion and job stress, affecting the “overexertion safety margin”. Exceeding optimal exertion levels increases the risk of WMSDs [56,57,65,66]. In our investigation, the risk related to exertion level (R4) is captured by Equation (11).

(11) $$R_{4}=\left\{\begin{array}{cc} 0, & \mathrm{if}\;CWL\in [0,PWL]\\ \frac{CWL-PWL}{MVC-PWL}, & \mathrm{if}\;CWL\in [PWL,MVC] \end{array}\right.$$

Lastly, adequate recovery time between tasks is essential to prevent WMSDs, as continuous repetitive motions and insufficient rest increase the risk [70,71]. To quantify this aspect, the risk related to recovery time (R5) is given by Equation (12).

(12) $$R5=\left\{\begin{array}{cc} \frac{RR-AR}{RR}, & \mathrm{if}\;AR\leq RR\\ 0, & \mathrm{if}\;AR>RR \end{array}\right.$$

### 3.3. Overall Contribution of Risk Factors to WMSDs

**Figure 3:**
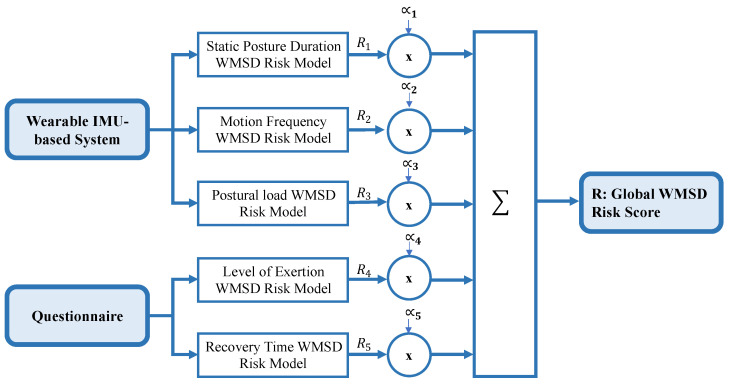
Simplified Illustration of the WMSDs Risk Model Proposed in this Study.

During work activity, risk factors combine contributing to the occurrence of WMSDs. This relationship is modeled in this study as stated by Equation (13) and Figure 3, assuming a perfect linearity between job safety and risk factors [56,57]. Alpha parameters ($\alpha_{i}$, *i* = 1:5) are constants used to adjust the influence of specific components of the model on the overall risk assessment. To our knowledge, limited to no prior research has studied the contribution of individual risk factors to the onset of WMSDs. Therefore, all risk factors are assumed to contribute equally to the development of overexertion and WMSDs ($\alpha_{i}=0.2$, *i* = 1:5).

(13) $$R=\alpha_{1}R_{1}+\alpha_{2}R_{2}+\alpha_{3}R_{3}+\alpha_{4}R_{4}+\alpha_{5}R_{5}$$

The results from the risk assessment models are presented in our proposed system’s user interface. This interface incorporates data visualization tools, such as pie charts and graphs, to illustrate the identified risks and their associated factors.

## 4. A Use Case for a Data-Driven WSMDs Risk Assessment Framework in Cable Manufacturing Industry

### 4.1. Experimental Setup

**Figure 4:**
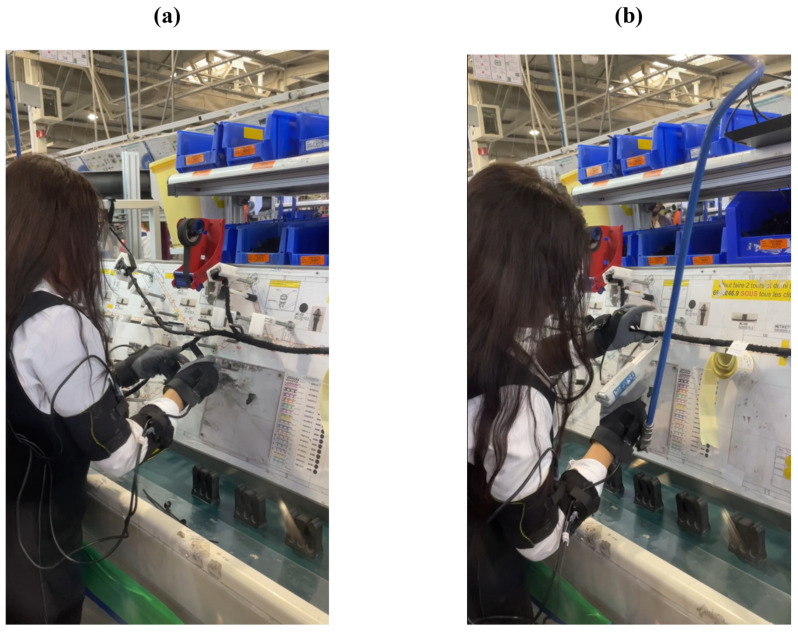
Real photos of the workstation main tasks: (**a**) Cable wrapping, and (**b**) Cable securing.

To evaluate the performance of our proposed system and risk assessment model, this study was carried out at a cable manufacturing facility, specifically at the final fixed workstation. The workstation mainly involves cable wrapping and securing (Figure 4a,b). Cable wrapping tape and a snap-on tool are used for these respective processes.

The workstation was located within a well-lit production area with moderate background noise from surrounding machinery. The work surface was a metal table with a conveyor belt for cable delivery. The ambient temperature was controlled at approximately 22 °C. The workstation incorporates an anti-fatigue mat. This cushioned platform is designed to alleviate stress exerted on the worker’s feet, legs, and lower back, thereby enhancing comfort and potentially mitigating the risk of WMSDs in the lower limbs. Regardless, this specific workstation has a high incidence of reported WMSDs. These reported WMSDs predominantly affect the upper limb joints, specifically manifesting as tendinitis of the shoulder, low back pain, and Carpal Tunnel Syndrome affecting the hands and wrists.

### 4.2. Participants and Data Collection Procedure

The research involved full-time workers at a cable manufacturing facility who had been assigned to the final fixed workstation for at least one year. The study included 10 female participants who were free of musculoskeletal disorders in the past six months and had no neurological or orthopedic conditions that could limit upper body mobility. Table 2 provides the demographic details of the participants.

After a brief overview of the study and signing a written consent, participants were equipped with IMU sensors, ensuring proper placement and attachment. Then, they were given brief training to practice the taping and securing tasks while wearing the sensors to ensure comfort with the equipment and task execution. Participants were instructed to perform 10 repetitions of their regular cable taping and securing tasks while wearing IMU sensors and to work at their normal pace and to maintain their typical work practices. The tasks involved standing in a still posture, repetitive upper limb movements for tape application, and frequent use of the snap-on tool to secure cable ends. Cables had the same thickness and weight, with an average taping and securing cycle lasting approximately 120 s. Data collection commenced at the start of the first cycle and continued until all cycles were performed.

The collected IMU sensor data were transferred in real-time to a secure computer for further processing and analysis. Simultaneously, videos have been recorded for visual reference. All recording sessions were performed at the same workstation to maintain consistency across the experiment. Upon the completion of the 10 cycles, sensors were carefully removed from participants. Participants were then given a brief debriefing, providing any additional information or instructions.

The study’s protocol was approved by the ISBM Ethics Committee (CERSVS/ISBM 018/2023) to ensure participant well-being and privacy.

### 4.3. Data Analysis and Interpretation

To assess the efficiency of the developed system for evaluating work cycle similarity and WMSD risk, a multi-step approach was employed. First, work cycles were first identified and stored within datasets. This facilitated inter-subject comparisons through calculations of standard deviation between a chosen representative cycle and the remaining cycles. Subsequently, an analysis of the inter-correlation between recorded samples was statistically performed using the intra-class correlation (ICC) across all joints and cycles.

Standard WMSD risk assessments, including RULA, REBA, Strain Index, and Rodgers Muscle Fatigue Analysis methods, were conducted alongside the proposed system. RULA provided frame-by-frame risk analysis at intervals of 3.5 s from work cycle videos, while REBA and Strain Index focused on task-level risks. Rodgers assessments identified joint-specific WMSD risks. The Rodgers Muscle Fatigue Analysis assigns a color-coded, three-digit score to specific joints. Each digit reflects effort level, duration, and frequency of movement, with higher scores indicating a greater risk of WMSDs.

The evaluation of these standard assessments was conducted using Ergofellow software 3.0 in collaboration with facility ergonomists to address subjectivity and ensure comparability of results. Risk scores were categorized into four action levels for comparison between the proposed method and the standard methods (Table 3).

The comparison between the system’s results and RULA involved the calculation of action-level distributions for statistical analysis. Subsequently, the results were downsampled to 0.29 Hz (3.5 s per sample) to visualize similarities and differences with RULA. Following this, the joint-specific risks identified by the proposed method were compared with the Rodgers Muscle Fatigue Analysis method. Lastly, the risk scores for cable placement, removal, wrapping, and securing tasks were calculated using the proposed method and were compared to REBA scores and the Strain Index.

## 5. Results

**Figure 5:**
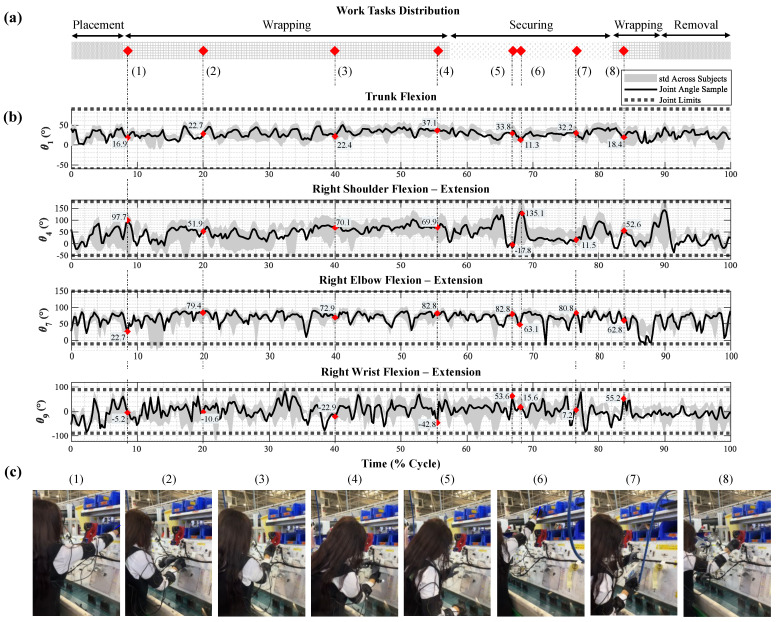
Fixed Workstation Work Cycle Analysis in Cable Industry (**a**) Tasks Distribution, (**b**) Joint Angles of Trunk, Shoulder, Elbow, and Wrist Flexions, (**c**) Observed Postures at Marked Timestamps.

Figure 5 illustrates a representative sample of joint angles observed during a single cable wrapping and securing cycle at the trunk, shoulder, forearm, and wrist joints. These data are part of a larger dataset of 100 cycles collected from 10 subjects. Figure 5a shows that the work task is distributed as 57% cable wrapping, 25% cable securing, and 18% cable placement and removal. To understand the variability in joint angles across all cycles, the standard deviation is calculated for each instance and shown in Figure 5b (shaded grey area). The participants exhibited similar joint patterns with few posture disparities, confirmed by an ICC=0.72±0.23. This suggests that, despite individual differences, the overall task demands exerted similar postural stresses on the musculoskeletal system. Photos corresponding to the marked timestamps in Figure 5a are shown in Figure 5c.

**Figure 6:**
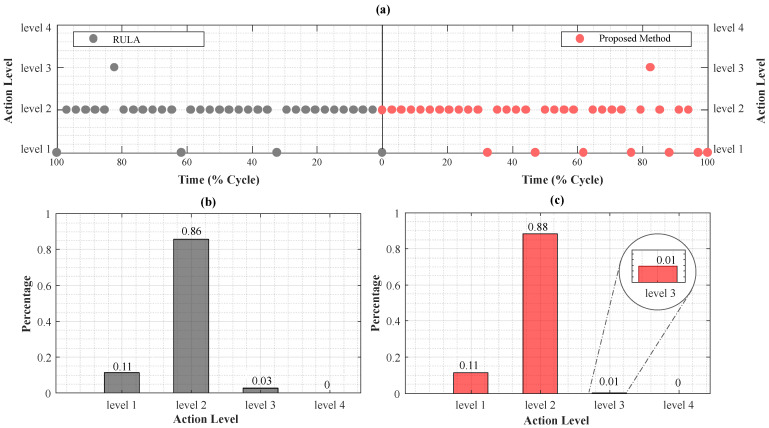
Cable Wrapping Action Levels: (**a**) Proposed vs. RULA, (**b**) RULA Distribution, (**c**) Proposed Method Distribution.

To evaluate the efficiency of the developed system in risk assessment compared to the standard RULA method, the time required for each approach was measured. The RULA analysis on a 120.5-s video resulted in approximately 35 observations and a completion time of 70 min. In contrast, the proposed system streamlined data processing and results generation, completing this step in approximately 2 min. Figure 6 compares risk assessments from both methods. The RULA scores in Figure 6b show 11% at action level 1, 86% at level 2, 3% at level 3, and 0% at level 4. Our system’s risk estimation demonstrates a similar distribution, with 11% at level 1, 88% at level 2, 1% at level 3, and 0% at level 4 (Figure 6c). As observed in Figure 6, results indicate that the majority of instances fall into action level 2. However, our approach showed a slightly higher percentage at this level (88% vs. 86%) and a slightly lower percentage at action level 3 (1% vs. 3%) compared to the RULA assessment. Notably, both methods found no instances at action level 4, indicating no immediate intervention is required for the studied tasks.

The presence of instances at action levels 2 and 3 necessitates further investigation and potential interventions to mitigate the risk of WMSDs. In this context, Table 4 shows the overall risks estimated by our proposed method averaged across all subjects.

Although the overall risk of WMSDs for the studied tasks is considered moderate 27.3 ± 2.4%, our method reveals moderate risks associated with prolonged static postures (R1=25±7.3%) and postural load (R3=14.1±2.6%), as well as significant regarding perceived exertion levels (R4=35±10.2%) and limited recovery time (R5=60%). Despite these findings, the task’s frequency (0.49±0.04 cycles/min) and cycle duration (122.4±20 s) initially appear low-risk (R2=0%).

To understand risk distribution across upper body regions, Table 5 reveals that the trunk exhibits the highest overall risk (35.6% ± 4.4%), likely due to prolonged static postures (71.3% ± 22%). This suggests a high potential for muscle fatigue and discomfort in this region. Elbows and forearms have substantial risk (30–34%), highlighting the impact of both static and awkward postures. Shoulders and Wrists show moderate risks, influenced by static postures (12–18%) and Postural Load Risk (19–22%), respectively.

To evaluate the effectiveness of our system, joint-specific WMSD risk assessment results were compared to those obtained from the Rodgers Muscle Fatigue Analysis, which identifies the most at-risk joints. As indicated in Table 6, the trunk is identified as the most critical joint with a score of 241 (action level 4), necessitating immediate intervention. Notably, the ergonomic expert assigned the highest risk value to the second digit (associated with duration of exertion) in the Rodgers Muscle Fatigue Analysis score. This aligns well with the high static posture risk (71.3% ± 22%) identified by our proposed method in Table 5. Interestingly, the Rodgers analysis assigns moderate risk (action level 2) to the remaining joints, which is consistent with the overall risk levels observed in Table 5.

Regarding the task-specific risk assessment, Table 7 summarizes the results provided by our method compared to the REBA and Strain Index. Across different methods, similar action-level classifications were observed for cable placement/removal and wrapping. Nevertheless, the results differed for cable securing, where our method assigns a lower action level (level 2) compared to the REBA and Strain Index that indicate level 3 with respective risk scores of 8 and 6.

**Figure 7:**
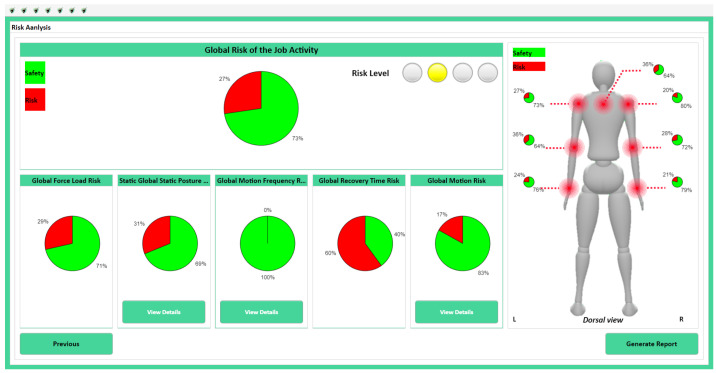
Illustration of the User-Interface Reporting WMSDs Risk Levels.

Within the user interface of our proposed wearable system, identified risks and their contributing factors are clearly highlighted, enhancing communication for ergonomic assessments, as illustrated in Figure 7.

## 6. Discussion

This study proposed a wearable IMU-based system for WMSD risk assessment in the upper body. Designed for industrial environments, this system utilizes IMU data to function independently of magnetic distortions. By optimizing sensor orientations and angle estimation algorithms, the system ensures data accuracy, resulting in a WMSD risk factors assessment well-suited for the demanding conditions of industrial settings. The system incorporates a user-friendly interface that facilitates seamless data collection, analysis, and visualization. This aligns with existing research emphasizing the importance of user interfaces in health monitoring systems, promoting user engagement and empowering healthcare professionals with objective insights for informed decision-making [75,76].

Based on motion capture technology, our system captures detailed biomechanical data on posture and joint angles throughout the work cycle. This approach provides a richer dataset compared to traditional observational methods like RULA, while also significantly reducing the time required for risk assessment. This difference likely explains the observed variations in risk scores between these methods [77,78]. While traditional methods have the advantage of not requiring specialized equipment, our method offers a deeper understanding of musculoskeletal stress through detailed biomechanical analysis.

Additionally, our method offers a comprehensive approach to workplace posture assessment compared to traditional methods. While it may identify fewer postures requiring immediate intervention due to its focus on detailed biomechanics, it highlights a significant prevalence of potentially risky postures that warrant attention. This proactive identification facilitated by our system allows for early intervention and the development of mitigation strategies, potentially preventing the onset of WMSDs in the long term.

Moreover, our method underscores the critical value of a multi-faceted approach that combines objective data, like average cycle duration and job frequency, with subjective worker feedback. This aligns with existing research highlighting the significance of addressing both the physical and perceived aspects of work to accurately assess and mitigate WMSD risks [79].

The findings of our study case specifically highlight the system’s effectiveness in ensuring targeted interventions in high-risk areas, such as the trunk, which is prone to prolonged static postures. The consistency between the proposed method and the Rodgers Muscle Fatigue Analysis reinforces the reliability of our risk assessment method. This leads to actionable recommendations like task redesign, adjustable workstations, or back supports to reduce identified risks [80,81,82,83]. Additionally, the analysis emphasizes the need for focusing on elbows, forearms, and wrists, prompting the adoption of ergonomic tools and adjustments in hand positions to mitigate the effects of static and awkward postures [81,84]. The moderate risk associated with wrist postures further underscores the importance of preventive measures like proper training and wrist rests [85,86].

By pinpointing joint-specific WMSD risks associated with each task, the proposed method allows for the development of customized interventions. For example, the system can identify the increased risk of shoulder tendinitis due to repetitive movements and awkward postures observed during cable wrapping tasks [87]. Similarly, it can highlight the link between prolonged standing, static trunk postures, and low back pain [88], alongside the association between repetitive hand and wrist movements with carpal tunnel syndrome [89]. This highlights the system’s potential for preventing WMSDs through early risk factor identification.

The task-specific risk assessment results suggest a general agreement between the proposed method, REBA, and the Strain Index for cable placement/removal and cable wrapping tasks. This indicates the effectiveness of the proposed method in these scenarios. However, for the cable securing task, the proposed method assigned a lower action level compared to the established methods. This difference suggests a potential underestimation of risk in the proposed method for this specific task.

A similar observation can be made regarding the overall risk level for the trunk in the joint-specific risk assessment. The proposed method appears to underestimate risk compared to the Rodgers Muscle Fatigue Analysis. This underestimation might be due to the equal weighting of all risk factors in the proposed method. For instance, assigning a higher weight to the duration of static posture could result in a higher risk score, potentially reducing the discrepancy between our method and the standard methods. This highlights the need for further research to refine the weighting of risk factors in relation to WMSD risks.

The study’s limitations, including the small sample size and focus on a single industry task, necessitate additional research with larger, diverse populations and various job settings to ensure robustness and generalizability. Future studies should also aim to enhance data precision and capture a wider range of movements, potentially integrating machine learning and decision-making algorithms to improve risk assessment accuracy and intervention strategies.

## 7. Conclusions

This research introduced a novel IMU-based system specifically tailored for assessing and managing Work-related Musculoskeletal Disorders (WMSDs). Utilizing advanced IMU technology, this system provides in-depth tracking and evaluation of movements and postures contributing to WMSDs, without relying on magnetometer inputs, ensuring its suitability for industrial settings where magnetic interference is common. Additionally, our system does not neglect the subjective assessment of the work situation, ensuring a comprehensive evaluation by combining objective data with workers’ personal experiences and perceptions. The implementation of a user-friendly interface streamlines the operational workflow, allowing for quick and efficient data collection, processing, and interpretation. This approach not only highlights the importance of merging ergonomic assessments with real-time biomechanical data but also significantly reduces the time required for analysis compared to traditional methods. The real-world application of our system in a cable manufacturing environment has proven its efficacy in identifying key motion and posture-related risk factors. Notably, our system pinpointed the specific risk factors that contribute to the previously reported WMSDs at the workstation, affirming and extending the findings of prior studies. This validation underscores the critical areas, particularly concerning trunk and upper limb postures, where workers are at an elevated risk of WMSDs, offering insights that surpass the depth provided by traditional assessment methods like RULA. Future efforts should focus on broader validation of this novel wearable IMU-based system across different work settings and populations to further establish its effectiveness and broaden its applicability in the field of occupational health. Additionally, exploring the relative influence of various WMSD risk factors could help refine the system’s risk assessment and intervention strategies for improved accuracy, enabling more targeted and personalized ergonomic interventions.

## Figures and Tables

**Table 1:** IMU-based upper body joint angles estimation using quaternion.

Sensor	w.r.t.	Formula	Euler Sequence	Joint Angles	
Trunk	Lower Back	$q_{LB}^{TR}=q_{LB}^{E}q_{E}^{TR}$	X Y Z	θ1 θ2 θ3	Trunk Flexion-Extension Trunk lateral Flexion Trunk Rotation
Arm	Trunk	$q_{TR}^{A}=q_{TR}^{E}q_{E}^{A}$	Y X Z	θ4 θ5 θ6	Shoulder Flexion and Extension Shoulder Adduction and Abduction Shoulder int. rot.
Forearm	Arm	$q_{A}^{F}=q_{A}^{E}q_{E}^{F}$	X Z Y	θ7 - θ8	Elbow Flexion and Extension Off-axis error Forearm pronation and supination
Hand	Forearm	$q_{F}^{H}=q_{F}^{E}q_{E}^{H}$	X Y Z	θ9 - θ10	Wrist Flexion-Extension Off-axis error Wrist radial-ulnar deviation

**Table 2:** Demographic Details of Participant Workers at a Cable Manufacturing Facility.

Attribute	Value
Sex	Female
Mean Age (years)	29.3 ± 4.8
Mean Height (cm)	161.7 ± 6.3
Mean Weight (kg)	61.4 ± 13.1
Average Experience (years)	2.1 ± 1

**Table 3:** Risk Score Ranges, Corresponding Action Levels, and Implications for RULA, REBA, Strain Index, Rodgers, and Proposed WMSDs Risk Assessment Methods.

Risk Assessment Method	Action Level			
	1	2	3	4
RULA	1–2	3–4	5–6	7
REBA	1–3	4–7	8–10	11–15
Strain Index	≤3	]3–5]	]5–7]	>7
Rodgers				
Proposed Method	≤20%	]20%–40%]	]40%–60%]	>60%
Implications	No immediate action required	Further investigation needed	Changes may be required soon	Immediate action required

Note: The colors in the table correspond to the risk levels based on Rodgers Muscle Fatigue Analysis: Purple indicates very high risk, Red indicates high risk, Yellow medium risk, and Green low risk, based on the three-digit scores which reflect effort level, duration, and frequency of movement.

**Table 4:** Overview of the Risk Scores from the WMSDs Risk Assessment Proposed Method.

Risk	Description	Mean% (Std)
R1	WMSDs Risk related to Static Postures Duration	25 (7.3)
R2	WMSDs Risk related to Job Frequency	0 (0)
R3	WMSDs Risk related to Postural load	14.1 (2.6)
R4	WMSDs Risk related to Level of exertion	35 (10.2)
R5	WMSDs Risk related to Recovery Time	60 (0)
R	Overall Risk of WMSDs	27.3 (2.4)

**Table 5:** Joint-Specific WMSDs Risk Assessment Results for Proposed Method during a Cable Industry Task.

Joint	Overall Risk (R)		Static Posture Risk (R1)		Postural Load Risk (R3)	
	Mean % (Std)	Action Level	Mean % (Std)	Action Level	Mean % (Std)	Action Level
Trunk	35.6 (4.4)	2	71.3 (22)	4	5.7 (3.4)	1
Right Shoulder	21.7 (3)	2	17.8 (12)	1	5 (1.7)	1
Left Shoulder	22.3 (3.4)	2	11.8 (8.14)	1	6.9 (3.8)	1
Right Elbow	33.9 (4.9)	2	33.6 (20.2)	2	31.4 (3)	2
Left Elbow	29.6 (3.8)	2	27.1 (18)	2	24.7 (7.5)	2
Right Wrist	24.1 (3.4)	2	5.3 (2.4)	1	19.9 (5.2)	1
Left wrist	24.7 (4.3)	2	7.1 (1.7)	1	22.3 (11)	2

**Table 6:** Results of the Joint-specific Rodgers Muscle Fatigue Analysis during a Cable Industry Task.

Joint	Rodger Score	Corresponding Color	Action Level
Trunk	241		4
Right Shoulder	212		2
Left Shoulder	212		2
Right Elbow	232		2
Left Elbow	232		2
Right Wrist	212		2
Left wrist	212		2

**Table 7:** Comparison of Task-specific WMSDs Risk Assessment for Proposed Method, REBA, and Strain Index during Cable Placement/Removal, Wrapping, and Securing.

Task	Proposed Method		REBA		Strain Index	
	Mean Score	Action Level	Score	Action Level	Score	Action Level
Cable placement/removal	25.6%	2	4	2	3.5	2
Cable wrapping	36.4%	2	5	2	4.25	2
Cable securing	38.2%	2	8	3	6	3

## Data Availability

Data are unavailable due to privacy restrictions.

## Abbreviations

The following abbreviations are used in this manuscript:

WMSDs	Work-related Musculoskeletal Disorders
IMU	Inertial Measurement Units
RULA	Rapid Upper Limb Assessment
REBA	Rapid Entire Body Assessment
